# Supplementary material for: Chromothripsis during telomere crisis is independent of NHEJ, and consistent with a replicative origin
Source: Genome Res. 2019 May;29(5):737–49. doi: 10.1101/gr.240705.118 (PMC6499312; doi:10.1101/gr.240705.118)
Supplement: Supplemental Material [file supp_gr.240705.118_Supplemental_file_1.zip › contigs/annotated_contigs/DB56/contig.2.DB56_length_758_mean_cov_16.2730870712.docx]

**DB56_length_758_mean_cov_16.2730870712**

```
TTTATTTTCTCATTGGTTTGGCTGGTTTTGTTTGATTTTAGATCCACCAACATGTTTGCCCATTTGGTTACTCACCTGTTCTTTCTTCT
 >chr6:3135282-3135686 + E=3e-228
CAAATGTTCCTTCTAAAAGCATTTATCTTCTCTTCTTCCTGAAGTTATTTTTAGAAGTTATTTAAGGGCAGCCTGTTAATGGTCAACTC

AGTTTTTACCAACAAGTGTCTTTTCTTTCATCCACCTTTTCTGTAAGTCTGTTTAGCTGAGTGTGGAAATGGAAGTGGAAAATCATTGT

CTCCCCCATTCTGCAGTCTTCTGCCTTCCATCCTTGCTGTTGAGAAGTTTCCTGTCCGTCTACGTGGTTCCTGCTTCGGAGTGATGTGT

CTTAACTCTCTGGCTGTTTTTCAGATTTTCTCCTTACCTTTGATATAT|ATGCAGATT|TGGATGGGCTGGGGTCACCAGTGCAGGACC
                                                          >chr6:3145088-3145432 + E=5e-
AGCCGCGTGTTCTGAGCCCAGGCCTGAGGGCCAGAGCCGGTGGGAAAGTCCATGTGGCACAGAGTTGGCATCTTAAGTTCGTGTCAACT
165
AAAGGGGAGGGAGAGGGGTTAAGAGCACCAGCAGGGGACTCAGACCTCGGTTGGAGTGCTGGTTTGGGTTTGAATGCTGGTGTGGGTTG

GAGTGCTGGTGTGGGTTGCAGTGCTGGTTTGGGTTGGAGTGCTGGTTTGGGGTGGAGTGCTGGTTCGGGTCCGAGTGCTGGTTCGGGTG

GAGTGCTGGTTCAGGGTGGAGTGCTGGTTCGGGGTGGAGTGCTGGTT|T
```
